# Combination of Acellular Nerve Graft and Schwann Cells-Like Cells for Rat Sciatic Nerve Regeneration

**DOI:** 10.1155/2014/139085

**Published:** 2014-07-09

**Authors:** Songtao Gao, Yan Zheng, Qiqing Cai, Zhansheng Deng, Weitao Yao, Jiaqiang Wang, Xin Wang, Peng Zhang

**Affiliations:** ^1^Department of Orthopedics, The Affiliated Tumor Hospital of Zhengzhou University, No. 127, Dongming Road, Zhengzhou, Henan 450008, China; ^2^Department of Radiology, The First Affiliated Hospital of Zhengzhou University, No. 1, Jianshe Road, Zhengzhou, Henan 450052, China; ^3^Department of Orthopedics, Xiangya Hospital of Central South University, No. 87, Xiangya Road, Changsha, Hunan 410008, China

## Abstract

*Objective.* To investigate the effect of tissue engineering nerve on repair of rat sciatic nerve defect.* Methods.* Forty-five rats with defective sciatic nerve were randomly divided into three groups. Rats in group A were repaired by acellular nerve grafts only. Rats in group B were repaired by tissue engineering nerve. In group C, rats were repaired by autogenous nerve grafts. After six and twelve weeks, sciatic nerve functional index (SFI), neural electrophysiology (NEP), histological and transmission electron microscope observation, recovery ratio of wet weight of gastrocnemius muscle, regenerated myelinated nerve fibers number, nerve fiber diameter, and thickness of the myelin sheath were measured to assess the effect.* Results.* After six and twelve weeks, the recovery ratio of SFI and wet weight of gastrocnemius muscle, NEP, and the result of regenerated myelinated nerve fibers in groups B and C were superior to that of group A (*P* < 0.05), and the difference between groups B and C was not statistically significant (*P* > 0.05).* Conclusion.* The tissue engineering nerve composed of acellular allogenic nerve scaffold and Schwann cells-like cells can effectively repair the nerve defect in rats and its effect was similar to that of the autogenous nerve grafts.

## 1. Introduction

Peripheral nerve injury is a common disease, the proliferation of glia and extraneural connective tissue in peripheral nerve defect may hinder the growth of anagenetic axon or misguide the growth cone of axon; thus, the anagenetic axon cannot reach the target organ or even results in the formation of neuroma at the nerve stumps [1]; therefore, the defect of nerve must be bridged by graft to induce the nerve regeneration. As the golden standard of the peripheral nerve defects therapy, the autologous nerve graft was effective for nerve repair, but the source of autologous nerve graft was limited and the application of this graft was limited by several elements such as the injury to donor for graft acquisition and the limited length for nerve defect repairment [2]. Other methods, such as nerve allograft, nonnerve tissue graft, and artificial nerve tissue graft, had been used, but their effect was not satisfactory [3].

This research intended to use the Schwann cells-like cells that differentiated from Adipose derived stem cells (ADSCs) as the seed cells and acellular nerve graft processed by chemical method as the three-dimensional cells scaffolds to construct the tissue-engineering peripheral nerve graft which used to repair the sciatic nerve defect of rats, thus providing the experimental base for the clinical application.

## 2. Materials and Methods

### 2.1. Animals

Twenty-five Sprague-Dawley rats and sixty F_344_ inbred rats (provided by Animal Experimental Center of Zhengzhou University) weighing around 200 g were used; all animals utilized in this research were cared for according to the policies and principles established by the animal welfare act and the NIH guide for the care and use of laboratory animals.

### 2.2. Methods

#### 2.2.1. Preparation of Acellular Nerve Scaffold

Twenty-five Sprague-Dawley rats were sacrificed by intraperitoneal anesthesia with 10% chloral hydrate solution (0.5 mL/100 g). After immersion sterilized in 75% alcohol for 15 min, bilateral sciatic nerves of 20 mm (total 50 nerves) were harvested for experiment under aseptic conditions. The isolated sciatic nerves were cleaned of external debris under a LZL-21 dissecting microscopy (Zhenjiang, Jiangsu, China). All nerves were used to prepare the acellular nerve graft according to Hudson method [4].

#### 2.2.2. Culture and Inducing Differentiation of ADSCs

Fifteen F_344_ inbred rats were sacrificed by intraperitoneal anesthesia with 10% chloral hydrate solution (0.5 mL/100 g). After immersion sterilized in 75% alcohol for 15 min, bilateral inguinal fat pad was harvested for experiment under aseptic conditions and minced after washing by PBS (phosphate buffer solution) and then dissociated with 0.075% collagenase type I for 90 min; the solution was passed through a 75 *μ*m filter to remove undissociated tissue and then neutralized by the DMEM of low glucose that contains 20% (v/v) fetal bovine serum and centrifuged at 1000 ×g for 8 min. The stromal cell pellets were resuspended in DMEM of low glucose that contains 20% (v/v) fetal bovine serum with 1% (v/v) penicillin/streptomycin solution, inoculated in 25 mL culture bottles in a density of 4 × 10^5^/mL. The medium was replaced after 3~4 d, and the nonadherent cells were removed. The cells were passaged with trypsin/EDTA and inoculated in 50 mL culture bottles when the ratio of cells fusion was up to 90%. Cultures were maintained in a 37°C incubator with 5% CO_2_; the fourth generation cells were induced to differentiation [5]. The adipose derived stem cells under subfused status of the fourth generation were used for induction according to Dezawa method [6] that induces the bone marrow stromal stem cells (BMSCs) to differentiate into Schwann-like cells in vitro. On the fifth day after induction, the ADSCs were digested using 0.25% trypsin and 0.02% EDTA and dropped in aseptic glass slides that were coated with polylysine after centrifugation and collection. Then the cells were incubated in 37°C for 2 h, washed by PBS for 5 min, dried and fixed by 4% paraformaldehyde, and stained by S-100 and glial fibrillary acidic protein (GFAP).

#### 2.2.3. Animal Experiment

Forty-five F_344_ inbred rats were randomly divided into three groups according to different methods of sciatic nerve defect repair; there were fifteen rats in each group. After intraperitoneal anesthesia with 10% chloral hydrate solution (0.5 mL/100 g); the rats were placed in a prone position and the skin was prepared. A standard longitudinal incision was made in the left gluteal region, and then the tissue between the subcutaneous and muscular layers was dissected. Following this, the main stem of left sciatic nerve was isolated at midthigh level and 10 mm nerve was removed under aseptic conditions; the nerve defect was repaired with different methods: group A, nerve defect was repaired using acellular nerve graft alone; group B, nerve defect was repaired with acellular nerve graft and Schwann cells-like cells that differentiated from ADSCs: when the ratio of cells fusion was up to 50%, the culture medium was replaced by 10 *μ*mol/mL culture medium that labeled by BrdU (5-bromo-2-deoxyuridine), a cell proliferation marker, cultures were maintained in a 37°C incubator with 5% CO_2_ for 24 h, 200 *μ*L Schwann cells-like cells that differentiated from ADSCs were suspended in Dulbecco's Modified Eagle Medium (DMEM) with 10% fetal bovine serum in a density of 1 × 10^7^/mL, and then 10 *μ*L cells suspension were point-injected into the 10 mm acellular nerve grafts by microinjector from one end to the other through the central axis of graft's cavity. After 4 h, the cells-scaffold combinations were placed in plates containing DMEM with 10% fetal bovine serum for 48 h at 37°C and 5% CO_2_ under saturated humidity for use; Group C, nerve defect was repaired with autogenous nerve that suture in situ. All the grafts were end-to-end anastomosis with the two ends of sciatic nerve defect by 9/0 nontraumatic suture needles and the normal right sciatic nerves were used as control.

*(1) Recovery Ratio of Sciatic Nerve Function Index (SFI%).* The SFI was calculated using the method described by Reynolds and Weiss [7]. At 6 w and 12 w following the surgery, footprints' record box made by us was used to measure the following variable quantity. The box was 8.5 cm wide and 50 cm long, with one door on the side of the box and a piece of 70 g white paper that is same size as the bottom of the box was placed in the bottom of the box. Rats that two hind paws had been dipped in carbon black ink walked from one end to the side door of the box, and three or four footprints of each foot were left on the paper. Three variable quantities of the experimental side feet and the normal side feet were measured as follows.

1. Print length (PL): the maximum print distance between heel to toe, the accuracy of length in millimeters and every time the maximum PL was used.
2. Toe spread (TS): the distance between 1st toe and 5th toe, the accuracy of length in millimeters and every time the maximum TS was used.
3. Intermediary toe spread (ITS): the distance between 2nd toe and 4th toe, the accuracy of length in millimeters and every time the maximum ITS was used.

The SFI was calculated in accordance with the formula described by Bain et al. [8] to work out SFI:

(1) SFI=−38.3(EPL−NPLNPL)+109.5(ETS−NTSNTS) +13.3(EIT−NITNIT)−8.8.

SFI = 0 was used as normal value, SFI = −100 was used as complete nerve transected, and recovery ratio of sciatic nerve function index (SFI%) was worked out by comparing the SFI of experimental side to that of the normal side.

*(2) Neural Electrophysiology (NEP).* 6 w and 12 w following the surgery, BL-410 biological function experiment system and related equipment were used to mensurate the motor nerve conduction velocity (MNCV) of bilateral sciatic nerve according to Foidart-Dessalle method [9] and the maximum amplitude of complex action potential (CMAP) of bilateral sciatic nerve, so the recovery ratio of them was calculated according to the following formulae:

(2) MNCV(ms)=Distance  between  two  points(m)Difference  value  in  latent  period  of  two  ends(s),Recovery  ratio  of  MNCV=the  mncv  in  operated  side  the  mncv  in  normal  control  side×100%,Recovery  ratio  of  CMAP=the  mean  amplitude  in  operated  side  at  different  times  the  mean  amplitude  in  normal  control  side  at  different  times × 100%.

*(3) Recovery Ratio of Wet Weight of Gastrocnemius Muscle.* After the neural electrophysiology experiment was finished, the gastrocnemius muscle of experiment and normal legs in all rats were taken out completely, and the wet weight of gastrocnemius muscle was weighed by electronic balance such that precision is 1/10000 grams. The recovery ratio of weight of gastrocnemius muscle was worked out by comparing the weight of gastrocnemius muscle of experimental side to that of the normal side.

*(4) Histological Observation.* After the above experiment, 3 mm nerve segment in the proximal part of nerve grafts and the parallel segment in the control nerve were fixed by 10% formalin fixation for 24 h; gradient alcohol dehydration and paraffin embedded, transverse, and longitudinal sections (5 *μ*m) of them were cut with a microtome. The tissue slices were deparaffinized with xylene 2 × 10 min and then rinsed in anhydrous alcohol 2 × 2 min and immersed in 95%, 80%, and 75% alcohol 1 min, respectively, and then rinsed in PBS 1 min. After staining by Harris hematoxylin for 5 min, the slices were rinsed in PBS for 1 min and differentiated with 10 mL/L hydrochloric acid alcohol 20 s. After rinsed in PBS for 1 min and returned to blue with 10 mL/L ammonia for 30 s, then rinsed in PBS for 1 min. After incubation in eosin staining 1 min, the slices were rinsed in PBS 30 s and dehydrated in 75% alcohol 20 s, in 80% alcohol 30 s, and in 95% alcohol 2 × 1 min. After rinsing in anhydrous alcohol 2 × 2 min and immersed in xylene 3 × 2 min, the slices were covered with general clarity gum. The longitudinal tissue slices from proximal part of nerve grafts in group B were deparaffinized with xylene 2 × 10 min and incubated by 3% H_2_O_2_ (30% H_2_O_2_ mixed with methyl alcohol in ratio of 1 : 10) for 10 min; after antigen repaired by microwave heating in citrate buffer solution, then the sealing liquid of 5% BSA was added and the first antigen (rabbit anti-rat antigen of BrdU and S-100 protein) was added under 37°C for 90 min, rinsed in PBS 2 × 2 min and the biotin-labeled secondary antibody (goat anti-rabbit antigen) was added that incubated under the room temperature for 20 min; SABC reagent was added and incubated under the room temperature for 20 min, and 3,3-diaminobenzidine (DAB) was used as ingrain agent. Myelinated fibers and myelin sheaths of regenerated nerve were observed under a CX31-12C04 light microscopy (Olympus, Japan). 3 mm nerve segment in the middle part of nerve graft and the parallel segment in the control nerve were taken to fix in 4% glutaraldehyde in PBS for 4 h at room temperature, then rinsed in PBS 3 × 3 min, fixed in osmic acid, rinsed in PBS 3 × 3 min, and dehydrated in ascending series of alcohol (50%, 70%, 90%, and 100%); Epon812 embedded, semithin section of 1 *μ*m was toluidine blue stained.

*(5) Transmission Electron Microscope.* 6 w and 12 w following the surgery, 3 mm nerve segments in the distal part of nerve grafts and the parallel segments in the control nerves were taken to fix in 4% glutaraldehyde in PBS for 4 h at room temperature, then rinsed in PBS 3 × 3 min, fixed in osmic acid, rinsed in PBS 3 × 3 min, and dehydrated in ascending series of alcohol (50%, 70%, 90%, and 100%); Epon812 embedded, ultrathin section of 20 nm was uranyl acetate and lead citrate stained; the regeneration of nerve was observed under transmission electron microscope.

*(6) Recovery Ratio of Quantity, Diameter, and Myelin Sheath Thickness in Regenerated Myelinated Nerve Fibers.* 6 w and 12 w following the surgery, the toluidine blue staining slices of bilateral nerve segments in each group were observed under light microscope that magnified 1000 times to count the quantity of myelinated nerve fibers in each field of vision and contrast the quantity of experimental side to the quantity of normal side to get the recovery ratio:

(3) Recovery  ratio  of  the  quantity  of  nerve  fiber  =Nerve  fiber  quantity  of  the  experimental  sideNerve  fiber  quantity  of  the  normal  side   ×100%.

Both the diameter and myelin sheath thickness in regenerated myelinated nerve fibers of each group were assessed in a double blind method with MOTICMED 6.0 digital medical imagine analysis system by pathological technician in our laboratory: the section of bilateral nerve segment in each group was observed under light microscope that magnified 400 times to count the quantity of myelinated nerve fibers in four fields of vision and record the diameter and myelin sheath thickness; the average was counted and the quantity of experimental side was contrasted to the quantity of normal side to get the recovery ratio:

(4) Recovery  ratio  of  the  diameter  of  nerve  fiber  =Nerve  fiber  diameter  of  the  experimental  sideNerve  fiber  diameter  of  the  normal  side   ×100%,Recovery  ratio  of  the  myelin  sheath  thickness  of  nerve  fiber  =Thickness  of  the  experimental  sideThickness  of  the  normal  side×100%.

### 2.3. Statistical Analysis

The results were analyzed by spss10.0 (SPSS Inc., Chicago, IL, USA); the main statistical methods include one-way analysis of variance (ANOVA) which was performed to determine the statistical significance between groups, and *t*-tests were used to determine whether the averages of the data sets were statistically significant. Values of *P* < 0.05 were considered to indicate a statistically significant.

## 3. Results

### 3.1. Identification of Schwann Cells-Like Cells That Differentiated from ADSCs

**Figure 1:**
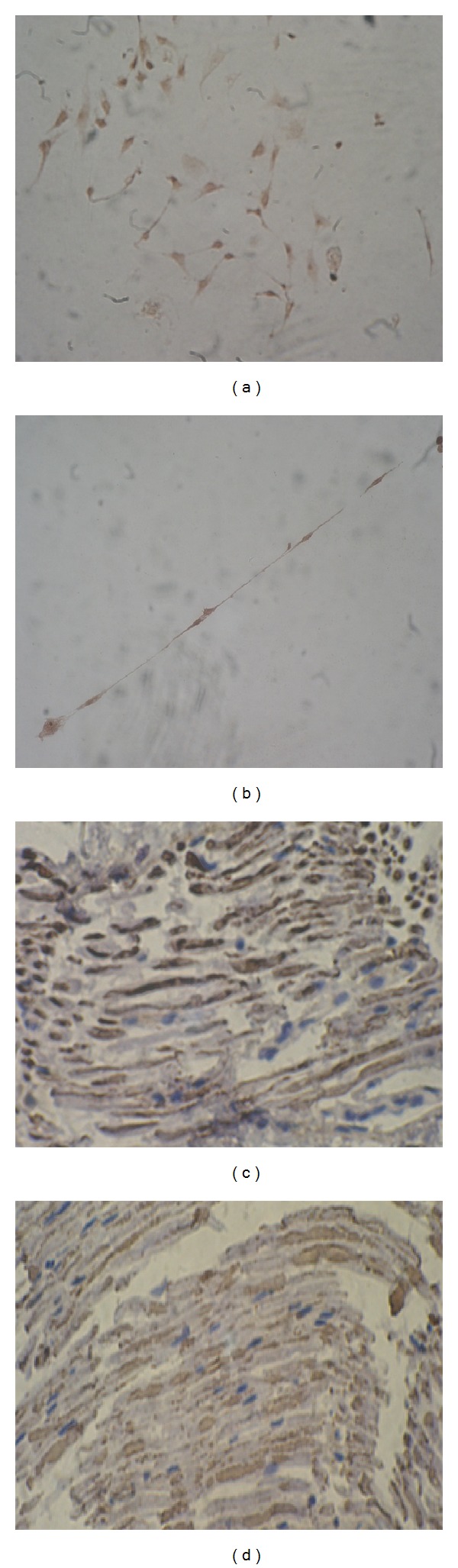
(a) Immunocytochemical staining of GFAP in Schwann cells-like cells that differentiated from ADSCs. (b) Immunocytochemical staining of S-100 in Schwann cells-like cells that differentiated from ADSCs. (c) BrdU and S-100 staining of longitudinal nerve slices from group B at 6 w, blue BrdU-labeled Schwann cells-like cells' nucleus, and brown S100-labeled staining myelin sheath were observed under microscope. (d) BrdU and S-100 staining of longitudinal nerve slices from group B at 12 w, blue BrdU-labeled Schwann cells-like cells' nucleus, and brown S100-labeled staining myelin sheath were observed under microscope.

After staining by S-100 and GFAP, the cytoplasm of the positive staining cells was yellow dyed; the morphology of positive staining cells was consistent with that of living cells observed under inverted microscope (Figures 1(a) and 1(b)).

### 3.2. Recovery Ratio of Sciatic Nerve Function Index (SFI%)

**Figure 2:**
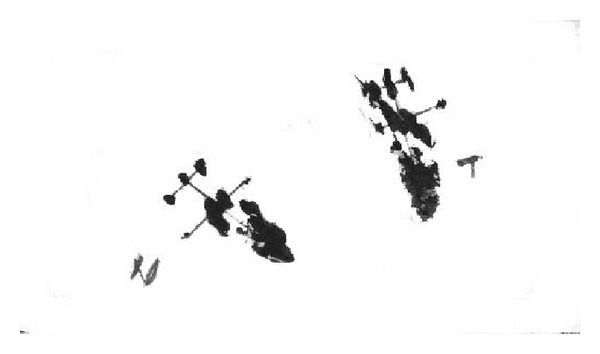
The footprint for sciatic nerve function index of rats.

6 w and 12 w following the surgery, the SFI% of groups B and C was superior to that of group A (*P* < 0.05), but the difference between groups B and C was not statistically significant (*P* > 0.05) (Table 1, Figure 2).

### 3.3. Neural Electrophysiology (NEP)

**Figure 3:**
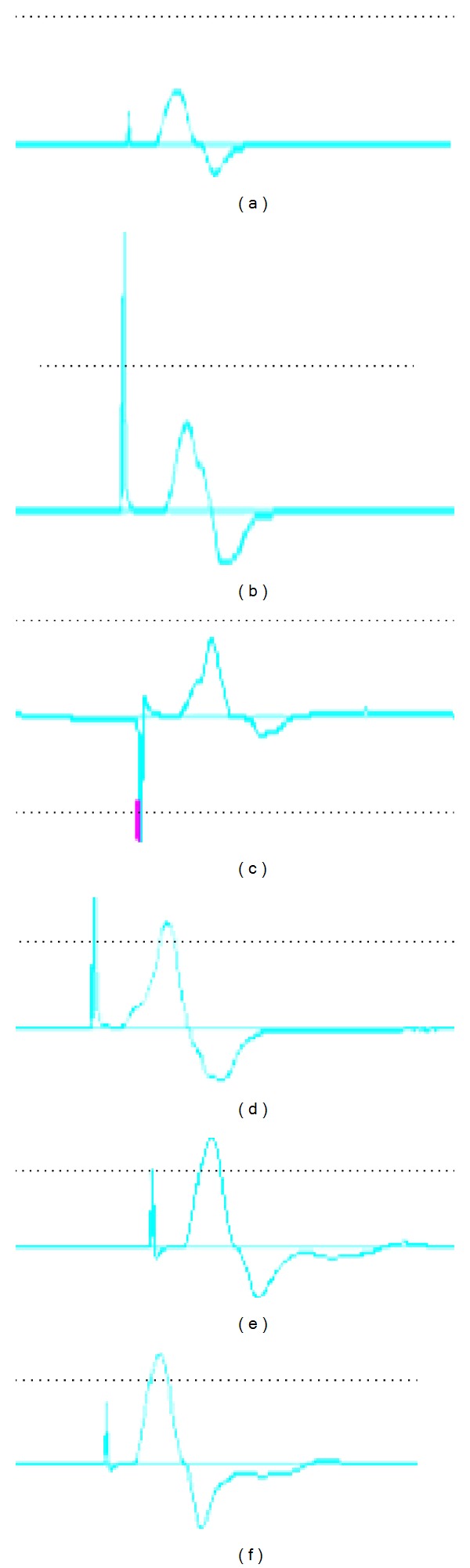
((a), (b), and (c)) Neural electrophysiology of group A (a), group B (b), and group C (c) at 6 weeks. ((d), (e), and (f)) Neural electrophysiology of group A (d), group B (e), and group C (f) at 12 weeks.

6 w and 12 w following the surgery, the recovery ratio of MNCV and CMAP of groups B and C was superior to that of group A (*P* < 0.05), but the difference between groups B and C was not statistically significant (*P* > 0.05) (Table 1, Figure 3).

### 3.4. Histological Observation

**Figure 4:**
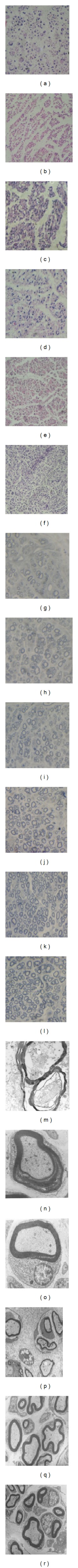
((a), (b), and (c)) HE staining of transverse nerve slices from each group at 6 w. The regenerated myelinated nerve fibers were sparse; distribution and diameter of the fibers were irregular, but there were no obvious lymphocytic infiltrates. ((d), (e), and (f)) HE staining of transverse nerve slices from each group at 12 w. (d) The number of regenerated myelinated nerve fibers in group A was increased; myelin was relatively dense and irregularly arranged. The number of regenerated myelinated nerve fibers in groups B (e) and C (f) was increased; myelin was dense and regularly arranged; there were blood capillary hyperplasia and no obvious lymphocytic infiltrates. ((g), (h), and (i)) Toluidine blue staining slices from each group at 6 w. (g) The myelin of regenerated myelinated nerve fibers in group A was sparse; the morphology and diameter were irregular. The myelin of regenerated myelinated nerve fibers in groups B (h) and C (i) was dense, but the morphology and diameter were irregular. ((j), (k), and (l)) Toluidine blue staining slices from each group at 12 w. (j) There were large numbers of regenerative and partly myelinated axon in group A and the diameter of them was homogeneous. There were large numbers of regenerative and partly myelinated axon in groups B (k) and C (l) and the diameter of them was homogeneous and myelin sheath was more thicker than group A. ((m), (n), and (o)) Transmission electron microscope from each group at 6 w. (m) The arrangement and thickness of regenerated myelin sheath in group A were irregular. (n) The arrangement of regenerated myelin sheath in group B was more regular than group A, but the thickness of myelin sheath was nonuniform. (o) The arrangement of regenerated myelin sheath in group C was more regular than group A and the thickness of myelin sheath was uniform. ((p), (q), and (r)) Transmission electron microscope from each group at 12 w. (p) The regenerated myelin sheath was thin and irregular, accompanied with Schwann cell proliferation. (q) In group B, the regenerated myelin sheath arranges compactly and thicker, accompanied with Schwann cell proliferation. (r) In group C, the regenerated myelin sheath arranges compactly and thicker than groups A and B, accompanied with Schwann cell proliferation and chondriosome, microtubule, and microfilament inside.

6 w following the surgery, in HE staining slices of three groups, the regenerated myelinated nerve fibers were sparse; the distribution and diameter of the fibers were irregular, but there were no obvious lymphocyte infiltration (Figures 4(a), 4(b), and 4(c)); 12 w following the surgery, in HE staining slices of three groups, the number of regenerated myelinated nerve fibers in group A was increased; although the myelin was relatively dense, it was irregularly arranged; the number of regenerated myelinated nerve fibers in groups B and C was increased, the myelin was densely and regularly arranged, and there was blood capillary hyperplasia and no obvious lymphocyte infiltration (Figures 4(d), 4(e), and 4(f)).

6 w following the surgery, in toluidine blue stained slices of three groups, the myelin of regenerated myelinated nerve fibers in group A was sparse, the morphology and diameter in group A were irregular, the myelin of regenerated myelinated nerve fibers in groups B and C was dense, but its morphology and diameter were irregular (Figures 4(g), 4(h), and 4(i)); 12w following the surgery, in toluidine blue stained slices of three groups, there were large numbers of regenerative and partly myelinated axon in group A and the diameter of them was homogeneous; there were large numbers of regenerative and partly myelinated axon in groups B and C and the diameter of them was homogeneous and the myelin sheath of them was more thicker than group A (Figures 4(j), 4(k), and 4(l)). 6 w and 12 w following the surgery, in stained slices from proximal part of nerve grafts in group B, some blue BrdU-labeled Schwann cells-like cells' nucleus and brown S100-labeled myelin sheath were observed under microscope (Figures 1(c) and 1(d)).

### 3.5. Transmission Electron Microscope

6 w following the surgery, the arrangement and thickness of regenerated myelin sheath in group A were irregular, the arrangement of regenerated myelin sheath in group B was more regular than group A, but the thickness of myelin sheath in group B were not uniform, and the arrangement of regenerated myelin sheath in group C was more regular than group A and the thickness of myelin sheath in group C was uniform (Figures 4(m), 4(n), and 4(o)). 12 w following the surgery in group A, the regenerated myelin sheath was thin and irregular, accompanied with Schwann cell proliferation; in group B, the regenerated myelin sheath was denser and thicker, accompanied with Schwann cell proliferation; in group C, the regenerated myelin sheath was denser and thicker than groups A and B, accompanied with Schwann cell proliferation and cytoplasmic organoids such as chondriosome, microtubule, and microfilament inside the nerve (Figures 4(p), 4(q), and 4(r)).

### 3.6. Recovery Ratio of Quantity, Diameter, and Myelin Sheath Thickness in Regenerated Myelinated Nerve Fibers and Recovery Ratio of Wet Weight of Gastrocnemius Muscle

6 w and 12 w following the surgery, the recovery ratio of quantity, diameter, and myelin sheath thickness in regenerated myelinated nerve fibers and recovery ratio of wet weight of gastrocnemius muscle of groups B and C were superior to those of group A (*P* < 0.05), but the difference between groups B and C was not statistically significant (*P* > 0.05) (Table 2).

## 4. Discussion

Schwann cells (SCs) are special gliocytes of peripheral nervous system. They have a close relationship with both the origin, development, morphology, function, and the regeneration of the peripheral nervous system. They provide protection and nutrition and a suitable microenvironment to the axon and promote the formation of myelin sheath. The prerequisite of injured nerve regeneration includes protection of the neurons, induction of axon growth, and guidance of the growth cone to identify the corresponding target organs and establish new functional synapses; Schwann cells play a crucial role in the above three steps [10]. Owing to the limited source and difficulty in cultivation of Schwann cells in vitro and the limited multiplication capacity of mature glial cells, how to acquire enough Schwann cells for transplantation has always been a serious challenge. Adipose derived stem cells (ADSCs) are multipotent stem cells originated from adipose tissue; they possess the capacity to differentiate not only into the traditional mesenchymal cell line [11, 12] but also into nonmesenchymal cell types such as skeletal muscle cells, cardiomyocytes, and nerve cells [13–18]. At the same time, ADSCs possess the advantage of extensive sources and superficial location of subcutaneous adipose tissue, and abundant cells are easily obtained from waste adipose tissue (about 1 × 10^9^ stem cell from 10 mL of adipose tissue) without complicated anesthesia and operation and lower incidence of infection to the donor. In addition, the culture conditions of ADSCs are not strictly required as BMSCs, and the ADSCs possess strong ability in proliferation (average passage time about 60 h) and stable multiplication ratio for 13~15 generation; furthermore, the proportion of senile and dead cells during the cell proliferation is low [19], so that ADSCs are gradually becoming an optimum selection for seed cell of tissue engineering.

Researches have shown that allograft nerve that is pretreated by cryopreservation, cryodesiccation, freeze thawing, and chemical process can reduce its antigenicity and avoid immunological rejection after transplantation. The application of acellular nerve allograft that is processed by chemical method in repair of the peripheral nerve defect has been reported by many scholars [3, 20]. Compared with other methods, the components such as cells and myelin which cause immunological rejection during nerve transplantation can be eliminated more thoroughly in acellular nerve allograft that is processed by chemical method, so that the possibility of immunological rejection will be reduced and the histocompatibility will be improved. Furthermore, the integrity of basement membrane which guides the regenerated axons to arrive at the target organs and contribute to the nerve regeneration will be reserved [21]. Thus, the extracellular matrix of acellular nerve allograft is a suitable scaffold that provides an analogical microenvironment for seed cell adhesion, growth, and differentiation into tissue-engineering peripheral nerve.

ADSCs can be induced and differentiated into Schwann cells-like cells in vitro [22, 23]; in this research, the differentiated cell expresses both S-100 and GFAP that exist in Schwann cells. At the same time, it have been observed that ADSCs can adhere and grow in the scaffold when they were implanted into the acellular nerve scaffold; during this period, the cytoactivity of them was similar to the cells in culture media (*P* > 0.05); it means good histocompatibility between cells and acellular nerve scaffold [24, 25]. In this research, when the tissue engineered peripheral nerve that is composed of acellular nerve scaffold and differentiated adipose derived stem cells or only acellular nerve scaffold were implanted to bridge the defect of sciatic nerve, there were no apparent lymphocytes infiltration in the regenerated nerve; it means good histocompatibility between acellular nerve scaffold and allogeneic host rats. 6 w and 12 w following the surgery, there were blue BrdU-labeled Schwann cells-like cells' nucleus and brown S100-labeled myelin sheath was observed in nerve grafts of group B; it means the acellular nerve scaffold provided a suitable living space for cells that were transplanted in vitro; furthermore, these cells have developed into functioning cells in vivo.

## 5. Conclusions

This study shows that tissue engineered peripheral nerve can repair the 10 mm defect of sciatic nerve effectively and a large number of myelinated nerve fibers pass through the nerve graft 6 w and 12 w following the surgery; it means that the tissue engineered peripheral nerve that composed of acellular nerve scaffold and differentiated adipose derived stem cells have successfully restored the innervation of target organs. The amount of regenerated nerve fibers of group B was obviously more than that of group A; the recovery ratio of sciatic nerve function index, wet weight of gastrocnemius muscle, and neural electrophysiology result were superior to those of group A. These results indicate that the recovery index of neural function agrees with the histological results; the probable reason lies in that the differentiation of ADSCs into Schwann cells in vitro increases the amount of Schwann cells, thus accelerating the migration and early regeneration of axons.

## Figures and Tables

**Table 1:** Comparison of SFI%, MNCV%, and CAMP% in 3 groups ($\overset{-}{x}\pm s$, *n* = 15).

	SFI%		MNCV%		CAMP%	
	6 w	12 w	6 w	12 w	6 w	12 w
Group A	46.32 ± 5.13*	72.73 ± 8.06*	42.54 ± 6.33*	69.34 ± 9.13*	40.52 ± 4.15*	68.24 ± 9.45
Group B	52.38 ± 7.12^#^	79.99 ± 10.33^#^	51.12 ± 8.15^#^	75.93 ± 5.95^#^	45.38 ± 4.12^#^	75.98 ± 10.99
Group C	54.32 ± 5.24	83.12 ± 5.89	54.65 ± 7.42	79.75 ± 3.99	48.76 ± 6.76	79.43 ± 6.76

*Compared with groups B and C, *P* < 0.05; ^#^compared with group C, *P* > 0.05.

**Table 2:** Comparison of the recovery rate of wet weight of gastrocnemius muscle, regenerated myelinated nerve fibers number, nerve fiber diameter, and the thickness of the myelin sheath in 3 groups ($\overset{-}{x}\pm s$, *n* = 15).

	Wet weight of gastrocnemius (%)		Nerve fibers number (%)		Nerve fiber diameter (%)		Thickness of the myelin sheath (%)	
	6 w	12 w	6 w	12 w	6 w	12 w	6 w	12 w
Group A	36.25 ± 4.22*	62.31 ± 3.96*	38.45 ± 6.28*	67.89 ± 11.87*	40.11 ± 4.32*	76.37 ± 9.38*	41.33 ± 4.58*	77.62 ± 7.98*
Group B	47.43 ± 6.43^#^	79.03 ± 5.66^#^	44.29 ± 7.52^#^	77.06 ± 11.54^#^	45.58 ± 4.87^#^	83.23 ± 6.32^#^	46.41 ± 4.35^#^	83.96 ± 8.31^#^
Group C	49.66 ± 6.48	80.43 ± 5.78	47.75 ± 5.98	81.25 ± 6.65	48.43 ± 5.76	87.87 ± 5.43	48.71 ± 6.65	87.56 ± 7.76

*Compared with groups B and C, *P* < 0.05; ^#^compared with group C, *P* > 0.05.
